# Diffusion-weighted MRI of bone marrow oedema, soft tissue oedema and synovitis in paediatric patients: feasibility and initial experience

**DOI:** 10.1186/1546-0096-10-20

**Published:** 2012-07-31

**Authors:** Henning Neubauer, Laura Evangelista, Henner Morbach, Hermann Girschick, Martina Prelog, Herbert Köstler, Dietbert Hahn, Meinrad Beer

**Affiliations:** 1 Institute of Radiology, Department of Paediatric Radiology, University Hospital Wuerzburg, Josef-Schneider-Straße 2, 97080, Wuerzburg, Germany; 2 Department of Paediatrics, University Hospital Wuerzburg, Josef-Schneider-Straße 2, 97080, Wuerzburg, Germany; 3 Department of Paediatrics, Klinikum am Friedrichshain, Landsberger Allee 49, 10249, Berlin, Germany; 4 Institute of Radiology, University Hospital Wuerzburg, Oberduerrbacher Str. 6, 97080, Wuerzburg, Germany

## Abstract

**Background:**

MRI has become the mainstay of diagnostic imaging in paediatric rheumatology for lesion detection, differential diagnosis and therapy surveillance. MR imaging of synovitis, in particular, is indispensable for early diagnosis and follow-up in arthritis patients. We used diffusion-weighted MRI (DWI) as a new imaging modality in comparison to standard MRI sequences to study bone marrow oedema, soft-tissue oedema and synovitis in paediatric patients.

**Methods:**

A total of 52 patients (mean age 11 ± 5 years) with bone marrow oedema (n = 31), soft-tissue oedema (n = 20) and synovitis (n = 15) were examined with transversal diffusion-weighted single-shot echoplanar imaging in addition to standard MR sequences (T2W TIRM, T1W pre- and post-contrast). Diffusion-weighted images were used for lesion detection and apparent diffusion coefficient (ADC, unit × 10^-3^ mm^2^/s) values were measured with ROI technique on ADC maps.

**Results:**

In 50 of 52 patients, DWI delineated the lesion of interest corresponding to pathological signal increase on standard sequences. Mean ADC was 1.60 ± 0.14 (range 1.38 - 1.99) in osseous lesions, 1.72 ± 0.31 (range 1.43 - 2.56) in soft tissue oedema and 2.82 ± 0.24 (range 2.47 - 3.18) for joint effusion (ANOVA p < 0.001). No significant difference in mean ADC was seen for inflammatory vs. non-inflammatory lesions. Relative signal intensity of oedema was similar for DWI and T2W TIRM. DWI visualised synovial restricted diffusion with a mean ADC of 2.12 ± 0.45 in 12 of 15 patients with synovitis.

**Conclusions:**

Diffusion-weighted MRI reliably visualises osseous and soft tissue oedema, as compared to standard sequences. DWI of synovitis is feasible in large joints and presents a novel approach to contrast-free imaging of synovitis. Whole-body DWI for chronic non-bacterial osteomyelitis should be evaluated in future studies.

## Background

In recent years, MRI has increasingly become the modality of choice for regional and whole-body imaging in patients with musculoskeletal disorders [1-3], and particularly so in paediatric patients [4,5]. MRI facilitates localization and differentiation of osseous and soft-tissue lesions and has been evaluated for therapy surveillance in patients with chronic inflammatory arthritis and chronic non-bacterial osteomyelitis (CNO), among others [4-6]. Diffusion-weighted MR imaging (DWI) is a novel functional imaging technique, which not only visualises restricted diffusion of water within oedematous cerebral tissue in acute stroke [7], but has also been applied to extra-neurological body regions for lesion detection and tissue characterisation with a particular focus on oncological imaging [8]. A limited number of studies have so far investigated the diagnostic utility of DWI in patients with musculoskeletal disorders [9]. To date, there is still a paucity of data on DWI of non-tumorous osseous and soft-tissue lesions in children. In our present study, we evaluated the feasibility in paediatric patients and the imaging characteristics of bone marrow oedema, soft-tissue oedema and synovitis on diffusion-weighted MRI.

## Methods

A total of 52 paediatric patients with non-tumorous musculoskeletal lesions underwent routine MRI with regional diffusion-weighted MRI (DWI) in addition to standard MRI sequences. The presence of neoplasm had been ruled out in all patients based on clinical history, imaging characteristics, biopsy and/or follow-up prior to inclusion in this retrospective study. Patients with proven or suspected musculoskeletal tumours were excluded from the study. All study work was conducted in accordance with the requirements of the Helsinki Declaration. The study work based on retrospective analysis of data from routine examinations does not require institutional review board approval at our institution. The treatment contract between patients and our university hospital covers the use of anonymized data for scientific purposes. Informed written consent was obtained from the legal guardians of all patients for all diagnostic and therapeutic measures.

Our study group included 23 females and 29 males with a mean age of 11 ± 5 years (median 13 years, range 12 months - 21 years). Indication for whole-body MRI was suspected CNO and follow-up for known CNO in 13 patients. All other patients had regional MRI for further diagnostic work-up of local symptoms after clinical evaluation, radiography and/or ultrasonography had failed to provide all required diagnostic information.

Sedation was required for five examinations of patients aged 1 to 6 years. The standard scan protocol comprised coronal T2W TIRM, pre-contrast T1W TSE and contrast-enhanced T1W TSE with fat saturation. Typical scanning parameters at 1.5 Tesla were: T2W TIRM (TR 7350 ms, TE 75 ms, TI 120 ms, flip angle 180°, field of view 380 mm, slice thickness 4 mm, in-plane spatial resolution 0.94 mm × 0.94 mm) and T1W TSE (TR 795 ms, TE 11 ms, flip angle 160°, field of view 380 mm, slice thickness 4 mm, in-plane spatial resolution 1.07 mm × 1.07 mm). Additional sequences were acquired in transversal or sagittal cross-sections as needed for diagnostic purposes. Thirteen patients had whole-body standard MRI for suspected bacterial or non-bacterial osteomyelitis. We used phased-array body coils for whole-body scanning in combination with the head coil for skull and feet. The remaining patients had regional MRI for local complaints with dedicated MRI coils (head, coil, knee coil, spine coil) or flex coils.

Based on signal alterations observed on conventional sequences, all patients underwent regional transversal single-shot diffusion-weighted echo-planar imaging at 1.5 Tesla (n = 50, Magnetom Avanto and Magnetom Symphony, Siemens Medical, Erlangen, Germany) and 3 Tesla (n = 2, Magnetom Skyra, Siemens Medical, Erlangen, Germany) during the same MRI examination. Typical scanning parameters at 1.5 Tesla included TR 4600 ms, TE 137 ms, flip angle 90°, fatsat, b-values of 0-50 s^2^/mm and 800-1000 s^2^/mm, bandwidth 976 Hz/pixel, echo spacing 1.17 ms, epi factor 128, slice thickness 6 mm, scanning time 41s to 2 min 50s with 2 to 6 averages. Isotropic diffusion-weighted images acquired at low and high b-values were used for lesion detection. Additional regional DWI sequences with coronal or sagittal slice orientations were acquired in eight patients in order to study feasibility of non-transversal DWI acquisition.

Among our 52 study patients, we identified 31 manifestations of bone marrow oedema, 20 manifestations of soft-tissue oedema and 15 cases of synovitis. One lesion per patient only was included for evaluation in each diagnostic category. The differential diagnosis of non-bacterial versus bacterial osteomyelitis was supported with biopsy and microbiological analysis of biopsy specimen.

ADC maps were automatically generated by the scanner software. The underlying algorithm is based on mono-exponential fit and per-pixel calculation according to the formula ADC = -1/(b_2_ - b_1_)ln(S_2_/S_1_), where S_1_ and S_2_ are the signal intensities with two different diffusion gradients b_1_ and b_2_[10]. Mean ADC_total_ was measured with ROI technique off-line on Syngo Plaza workstations (Siemens Medical, Erlangen, Germany), using one circular ROI in bone marrow oedema and soft-tissue oedema and calculating the average value of three small circular ROIs for synovial ADC. Size of the circular ROI equalled the smallest transversal lesion diameter in small foci < 10 mm and was chosen 10 to 15 mm in diameter in larger lesion. Mean ROI area and standard deviation were 1.1 ± 0.5 cm^2^ for bone marrow lesions, 0.9 ± 0.5 cm^2^ for soft-tissue lesions and 5.2 ± 2.9 mm^2^ for synovitis.

All DWI measurements were performed by the same board-certified radiologist with 4 years of experience in extra-neurological diffusion-weighted MRI, who was blinded to clinical data. As a measure of relative signal intensity and detectability, we determined signal intensities of pathological lesions and adjacent normal tissue with ROI techniques corresponding to ADC measurement, comparing altered signal of oedematous bone marrow with normal bone marrow, soft-tissue oedema with adjacent unaffected soft tissue, as well as synovial signal alterations with joint effusion and periarticular soft tissue. For signal quantification, we employed a signal intensity (SI) ratio calculated as SI = SI_pathological_ / SI_normal_. Commercially available software (3D fusion, version A30, Siemens Medical, Erlangen, Germany) was used for semi-automatic image fusion of standard sequences with DWI image sets.

### Statistical analysis

Normally distributed data is presented as mean ± standard deviation. Between-groups comparison was performed with the independent sample t test for variables following normal distribution and the Mann-Whitney test for variables deviating from normal distribution. Kendall's W was calculated for between-groups comparison of more than two groups. For analysis of differences in lesion size, the difference of the longest transversal lesion diameter on DWI and T1W post-contrast images was tested with a one-sample t test against a test value of zero. A p value < 0.05 was considered as indicating statistical significance. Analyses were performed with the PASW (SPSS) Statistics 18 software package (SPSS Inc., Chicago, USA).

## Results

All 52 MRI studies yielded diagnostic image quality. Forty-one patients showed bone oedema and/or soft-tissue oedema on standard MRI (Table 1). Among these were 4 patients with concurrent synovitis, while synovitis only was observed in the remaining 11 patients. Two post-contrast T1W studies showed moderate motion artefacts. In one patient with pedal phalangeal bone marrow oedema and in one patient with carpal synovitis, the lesion of interest could not be identified on DWI due to distortion artefacts, while all other lesions were detectable both on standard sequences and on DWI.

**Table 1:** Distribution and aetiology of bone marrow and soft-tissue oedema on a per-lesion basis in 41 patients

	**Bone marrow oedema**	**Soft-tissue oedema**
**number of lesions**	**31**	**20**
**localisation**		
head/neck	0	3
arm	0	1
spine	1	2
pelvis	5	2
upper leg	5	3
lower leg	13	8
foot	7	1
**inflammatory**	**14**	**6**
infectious	6	5
non-infectious	8	1
**non-inflammatory**	**17**	**14**
trauma	15	7
post-operative	1	6
others	1	1

### Bone marrow oedema

**Figure 1:**
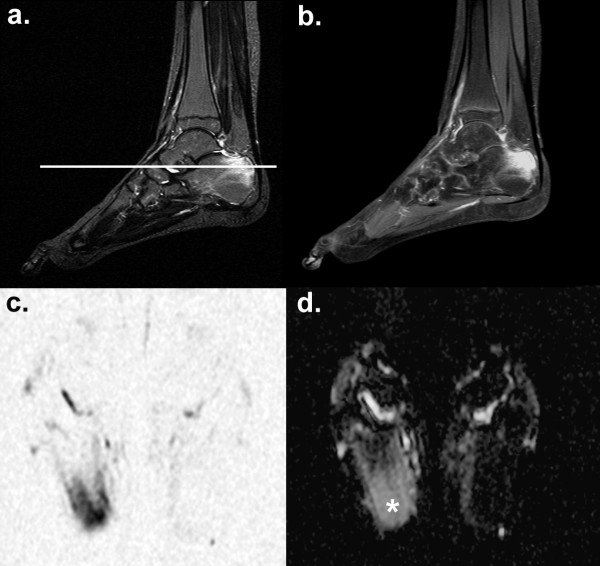
**Bone marrow oedema in chronic non-bacterial osteomyelitis (CNO).** A 12-year-old girl with multifocal CNO manifestations (rip bones, proximal femur, lower leg, metatarsal) shows intraosseous and paraosseal oedema on sagittal T2W TIRM **(a)**, corresponding contrast enhancement on sagittal T1W TSE FS **(b)** and correlating signal changes on transversal DWI in the right calcanear tuber **(c)**. The white line in (a) marks the cross-sectional plane of the DWI slice. Mean ADC of bone marrow oedema (white asterix) was 1.64 × 10^-3^ mm^2^/s **(d).**

**Figure 2:**
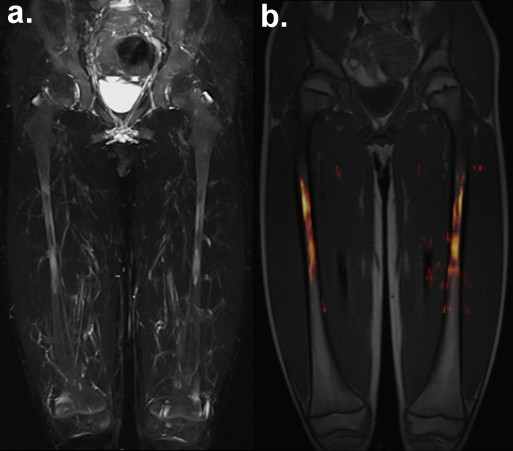
**Visual presentation of femoral bone marrow oedema with T2W TIRM and DWI.** Symmetrical bone marrow oedema of the femoral diaphysis in an 8-year-old boy with CNO. Visualisation with maximum intensity projection (MIP) of coronal T2W TIRM **(a)** and 3D image fusion of colourised transversal DWI b = 800 overlay on coronal pre-contrast T1W TSE **(b).**

**Figure 3:**
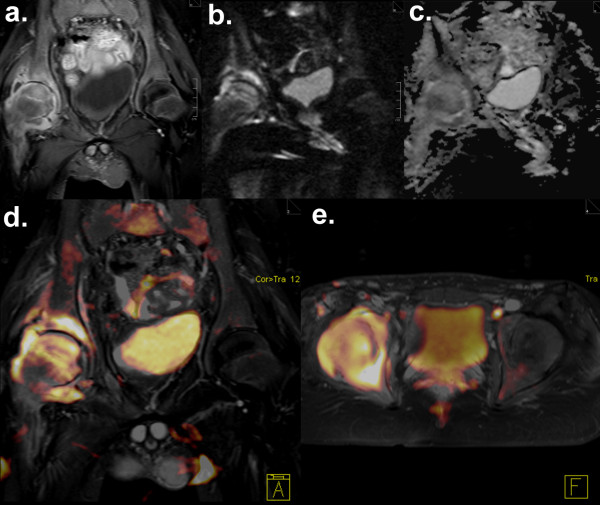
**Bone marrow oedema and soft tissue oedema in tuberculous coxarthritis.** Confirmed tuberculous coxarthritis of the right hip in a 15-year-old boy. Post-contrast T1W TSE FS shows severe coxarthritis **(a)** with osteomyelitis of the acetabulum and the femoral head and soft-tissue oedema in the right gluteus muscle. No pathological signal changes are seen in the left hip joint. Moderate distortion artefacts are noticeable on DWI with primary coronal acquisition **(b)**, while signal changes corresponding to (a) remain clearly visible. ADC values of the inflammatory osseous and soft-tissue lesions measured on the ADC map **(c)** ranged between 1.60 and 1.71 × 10^-3^ mm^2^/s. Image fusion of colourised DWI b = 800 data acquired with standard transversal DWI was performed with coronal T2W TIRM **(d)** and transversal post-contrast T1W imaging **(e).**

Inflammatory bone marrow oedema included bacterial osteomyelitis (n = 6), non-bacterial osteomyelitis in patients with CNO/CRMO (n = 6) and enthesitis of unknown aetiology (n = 2) (Figures 1, 2, 3). Non-inflammatory bone marrow oedema occurred secondary to blunt trauma (n = 15), post-operatively after bone biopsy (n = 1) and in a patient with Camurati-Engelmann syndrome.

Lesion size ranged from 6 mm to 67 mm on standard MRI and 8 mm to 71 mm on DWI (mean diameter 27 mm vs. 24 mm, p < 0.05). The mean signal intensity ratio of bone marrow oedema to normal bone marrow was 5.8 ± 3.5 for DWI at high b-values, 5.6 ± 5.9 for T2W TIRM and 3.6 ± 1.9 for fat-saturated contrast-enhanced T1W (Kendall's W test p = 0.304). Mean ADC of bone marrow oedema was 1.60 ± 0.14 × 10^-3^ mm^2^/s (range 1.38 to 1.99 × 10^-3^ mm^2^/s). No significant difference in mean ADC was observed for inflammatory versus non-inflammatory oedema.

### Soft tissue oedema

Inflammatory soft-tissue oedema (Figure 3) occurred secondary to soft-tissue abscess or joint empyema (n = 5) and adjacent to a focus of enthesitis (n = 1). Non-inflammatory soft-tissue oedema was of traumatic (n = 7) or post-operative (n = 6) origin, while one patient had lymphoedema of the lower extremity.

Lesion size was measured as 4 mm to 45 mm on standard MRI and 4 mm to 46 mm on DWI with a mean diameter of 21 mm for both sequences. The mean signal intensity ratio of soft-tissue lesions to adjacent normal soft tissue was 4.4 ± 2.0 for DWI at high b-values, 4.6 ± 2.5 for T2W TIRM and 2.3 ± 0.9 for fat-saturated contrast-enhanced T1W (Kendall's W test p < 0.001). Mean ADC of soft-tissue oedema was 1.72 ± 0.31 × 10^-3^ mm^2^/s (range 1.43 to 2.56 × 10^-3^ mm^2^/s).

### Synovitis

**Figure 4:**
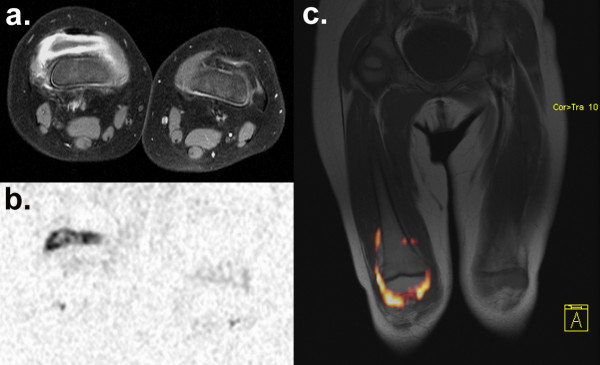
**Diffusion-weighted imaging of synovitis.** Gonarthritis of the right knee in a 2-year-old girl with juvenile idiopathic arthritis (JIA). Marked synovial thickening and contrast enhancement accompanied by joint effusion are seen on transversal T1W TSE FS **(a)**. DWI at b = 1000 with inverted gray scale depicts synovitis based on restricted diffusion on a corresponding transversal section acquired with 2 averages and 41s scan time **(b)**. Joint effusion shows low signal on DWI and can be distinguished from the surrounding layer of synovitis. The unaffected left knee does not show inflammatory signal alterations on contrast-enhanced T1W imaging, nor on DWI. Overlay of colourised DWI b = 1000 data on coronal pre-contrast T1W TSE **(c)** with semi-automatic 3D image fusion.

**Figure 5:**
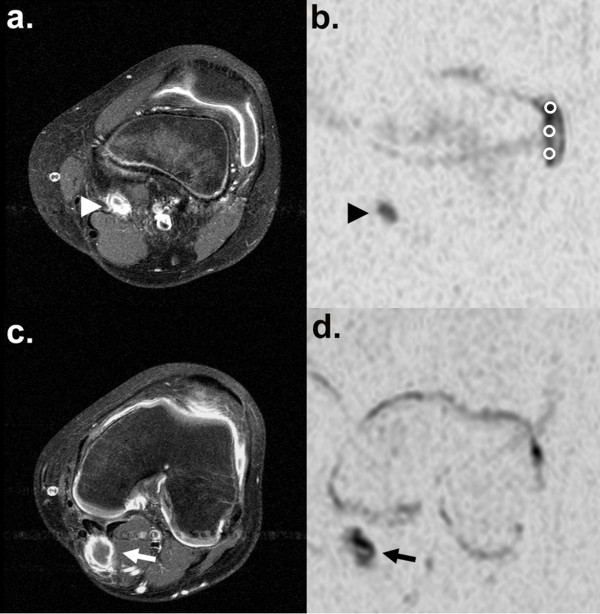
**Diffusion-weighted imaging in a patient with gonarthritis and a popliteal cyst.** Arthritis of the left knee in a 14-year-old boy with JIA. Post-contrast T1W **(a)** and DWI b = 1000 with inverted gray scale **(b)** both show synovial signal changes, layered signal of the parapatellar lateral recessus in the presence of effusion and reactive popliteal lymphadenopathy (arrow head). With 6 averages and 2 min 50 s scan time, DWI signal and image quality are substantially improved, as compared to Figure 4. A caudal cross-section of the same knee joint depics a Baker cyst in the medial popliteal fossa, also showing signs of synovitis (arrow) on both post-contrast T1W **(c)** and DWI **(d)**. ADC measurement with three small circular regions of interest (ROI) is demonstrated in (b).

Fifteen patients showed synovial thickening and contrast enhancement on T1W fat-saturated sequences and were diagnosed with synovitis. Thickening of the synovial layer was measured as 2-3 mm in all but one patient with omarthritis, who showed significant synovial proliferation up to 5 mm in thickness. Synovitis was observed on standard MRI in the glenohumeral joint (n = 1), elbow (n = 1), carpus (n = 1), hip (n = 1), knee (n = 7), ankle joint (n = 3) and as tenovaginitis of the flexor hallucis longus and tibialis posterior tendon sheath (n = 1). On DWI, synovial signal could not be discriminated from background signal in one patient with carpal arthritis. We observed signal elevation on DWI b = 800-1000 corresponding to synovial thickening and synovial contrast enhancement in all five synovitis patients without joint effusion. In the presence of effusion, we could distinguish between synovitis and effusion in seven of nine patients (Figures 4, 5), based on the DWI with high b values and the ADC map. In two patients, synovial signal and effusion could not reliably be distinguished on DWI. In summary, DWI visualised synovial inflammation based on restricted diffusion in 12 of 15 patients with synovitis.

Mean ADC of inflamed synovia was 2.12 ± 0.45 × 10^-3^ mm^2^/s, ranging from 1.36 × 10^-3^ mm^2^/s in a patient with tuberculous coxarthritis to 2.46 × 10^-3^ mm^2^/s in juvenile idiopathic arthritis. Median ADC of joint effusion was 2.77 × 10^-3^ mm^2^/s (range 1.35 - 3.18 × 10^-3^ mm^2^/s). The mean signal intensity ratio of inflamed synovia to normal bone marrow was 9.4 ± 6.3 for DWI at high b-values and 4.9 ± 1.7 for fat-saturated contrast-enhanced T1W (independent sample t test, p = 0.038). Signal intensity of inflamed synovia, compared to joint effusion, showed a median signal ratio of 1.6 for DWI at high b-values and 5.4 for fat-saturated contrast-enhanced T1W (independent sample t test, p = 0.008).

Eight examinations with additional sagittal and coronal DWI scans all showed marked artefacts which severely compromised diagnostic image quality in 5 patients. An example of coronal DWI of the pelvis with moderate artefacts is shown in Figure 3.

## Discussion

We present first results on feasibility and imaging characteristics of DWI in a paediatric cohort with a variety of inflammatory and traumatic musculoskeletal lesions. Our study results provide evidence that regional DWI can reliably detect and characterise such lesions in paediatric patients. The ADC values of bone marrow oedema and soft tissue oedema in our patient cohort compare well with data available from earlier pioneering studies on musculoskeletal DWI [11,12]. DWI offers new functional parameters, based on tissue diffusivity, for imaging of rheumatic disease in addition to standard assessment of oedema on T2W sequences and to contrast enhancement on contrast-enhanced T1W MRI. Future studies should assess whether DWI-derived measures provide, for example, added value for further differentiation of musculoskeletal lesions, as shown for benign vertebral fracture versus tumour infiltration in adults [12], and for therapy surveillance in patients with rheumatic disorders. With high-end MRI hardware, whole-body DWI has become technically feasible and should be evaluated in comparison to standard MRI sequences, as demonstrated for oncological disease [13].

For our present study, we focussed on lesion characterisation with regional DWI scans and transversal image acquisition. According to our experience, and as confirmed by our study results, primary coronal and sagittal acquisition frequently suffer from severe distortion and "ghost" artefacts and often result in non-diagnostic image quality, depending on patient body volume, patient position in the scanner and body region scanned.

Our quantitative data on relative signal intensity show favourable results for DWI in comparison to T2W TIRM and post-contrast T1W. Delineation of bone marrow oedema and soft-tissue oedema based on signal elevation is comparable for DWI and T2W TIRM. In terms of signal intensity, DWI may be superior to contrast-enhanced T1W in soft-tissue oedema. As diffusion-weighted images are characterised by an inherent strong suppression of the anatomical background, areas of restricted diffusion stand out from the surrounding structures and thus would be even more easily detected. Future studies should compare detection rates on DWI and standard sequences as well as learning curves for observers with different levels of experience.

Our report on sensitivity of DWI to synovial inflammation is novel, to our best knowledge. Extensive literature review revealed but one previous report on adult patients by Agarwal et al. who successfully employed diffusion-tensor imaging (DTI) for visualisation of synovitis in correlation with biomarkers of inflammation and who demonstrated therapy effects on synovial diffusivity upon follow-up [14]. Similar to DWI, DTI is also based on altered diffusivity of extracellular water in biological tissues. However, DTI provides information on the vectrality, that is, the spatial orientation of tissue structures based on the diffusion tensor, which is derived from at least six sets of diffusion-weighted images in non-collinear spatial directions [15]. In our present study we employed a standard DWI sequence with three orthogonal diffusion gradients, two b-values and very short scanning time of 41 s to 2 min 50 s, depending on the number of averages measured. ADC values in patients with synovitis showed a large inter-individual variation and a significant overlap between synovia and effusion in our study, most likely due to partial volume effects resulting from the limited in-plane resolution and slice thickness. Selection of b-values may also exert a particular influence on ADC values measured in inflamed synovial tissue. Tissue diffusivity measured at low b-values is affected by tissue capillary microperfusion in addition to extracellular diffusion, while perfusion does not exert a noticeable effect on DWI at high b values. Therefore, ADC signal attenuation curves derived from multiple b-value measurements show a biexponential slope [16]. In synovitis, synovial perfusion, vascular density and capillary permeability are markedly increased, which allows for quantification of inflammation and of therapy effects with the help of biomathematical modelling [17]. Therefore, a shift toward high total ADC can be expected for inflamed synovia, depending on the degree of hyperperfusion, if low b-values < 100 s^2^/mm and a mono-exponential model are used. Furthermore, synovial haemosiderin deposition observed in arthritic joints can affect T2W and gradient-echo sequences [18] and may also cause signal changes on DWI due to susceptibility. Therefore, acquisition of diffusion-weighted images with several b-values may prove helpful to better distinguish between synovitis and effusion and warrants evaluation in future studies. DWI with more than two b-values allows decomposition of the slow and the fast ADC component, which has been proposed as a useful tool in therapy monitoring of tumours [16]. Novel diffusion-weighted imaging techniques may also help to overcome disadvantages of standard single-shot echoplanar DWI in terms of imaging artefacts and signal-to-noise ratio [19].

Presently, MR imaging of synovitis largely relies on contrast-enhanced T1W images after intravenous administration of gadolinium-containing contrast agents [20]. However, some patients cannot, or do not want to, receive intravenous contrast medium for a history of allergic reaction or impaired renal function. As detection and quantification of synovitis on MRI is extremely helpful for early diagnosis and therapy surveillance in patients with chronic inflammatory arthritis, recent research efforts have focussed on native MR imaging techniques for synovitis without the need of intravenous contrast injection, such as arterial spin labelling [21]. DWI as employed in our study presents another innovative approach with promising first results and appears particularly appealing with respect to paediatric patients for merits such as short scanning time and relative insensitivity to patient movement.

### Limitations

Our study presents first experiences and proof-of-concept work, which implies important limitations to the interpretability of data in terms of study design, size of cohort and the absence of healthy controls. All DWI were acquired during clinical routine with standard scanning equipment and only limited time slots available for DWI scanning to assure patient comfort and quality of routine diagnostic imaging. Our patient group was examined with three different MR scanners of the same manufacturer at two different B0 field strengths. Scan protocols were designed with particular emphasis on comparability. Although there are no systematic studies on how DWI and ADC values depend on scanner type and technical setup, a certain interference of such variables with our study results cannot be ruled out completely. An earlier study, we did not find significant differences between-scanner differences in mean ADC [22].

SS-EPI DWI in its present technical application is prone to distortion artefacts, particularly with coronal and sagittal acquisition, and may not be applicable to small acral anatomical structures. Quantitative analysis of relative signal intensities and of ADC values with manual ROI selection is certainly affected by subjective judgement and could be improved with semi-automatic ROI selection. Considering the limited in-plane resolution and the slice thickness of 6 mm used for our DWI sequence, a certain influence of partial volume effects on ADC values can be expected, particularly in small lesions and in synovial measurements. Our preliminary results warrant verification in a more standardised and controlled research environment.

An issue not addressed in our study is the relation between bone marrow cellularity and bone marrow signal on diffusion-weighted MRI [23]. Extent and distribution of physiologically hypercellular haematopoietic bone marrow in children widely vary with age and may affect detectability and ADC of bone marrow lesions. To our knowledge, there is no systematic data on physiological DWI signal distribution in the bone marrow of children differentiated by age and anatomical localisation. The DWI signal of oedematous lesions may differ depending on whether the lesion is located within fatty or cellular bone marrow. For our study, we measured ADC of the focal bone marrow oedema only, which may be influenced by the properties of the surrounding intramedullar tissue. Signal intensity of lesions, however, was measured relative to adjacent normal-appearing bone marrow, so that the signal intensity ratios include the potential influence of different bone marrow cellularity. A more detailed analysis of bone marrow cellularity and distribution in relation to age is beyond the scope of the present study.

Finally, our data was collected from a relatively small, heterogenous group of patients, therefore the results of our statistical analysis need to be considered as preliminary.

In summary, we consider DWI a powerful tool for musculoskeletal imaging. The option of contrast-free scanning certainly is of clinical significance. Secondly, DWI provides additional information to differentiate tumorous from inflammatory musculoskeletal lesions and abscess [22]. DWI is less sensitive to motion artefacts, than are standard sequences. Fast diffusion-weighted image acquisition with EPI may thus be diagnostic in children who do not cooperate sufficiently for acquisition of standard sequences. Once whole-body DWI is established for routine scanning, it will facilitate detection and differentiation of neoplastic and inflammatory lesions, radiation- and contrast-free, relatively insensitive to patient motion (free-breathing acquisition, compliance in paediatric patients), with about 20 to 30 minutes examination time for a whole-body scan. Finally, our first experiences with diffusion-weighted imaging of synovitis are encouraging and deserve further evaluation.

## Conclusions

Diffusion-weighted imaging reliably visualises bone marrow oedema and soft tissue oedema, as compared to standard sequences, and may facilitate whole-body imaging for rheumatic disease in children in the near future. DWI presents a novel approach to imaging of synovitis free of i.v. contrast medium. Our findings warrant further studies to improve image quality, to evaluate the relation between restricted synovial diffusivity and clinical disease activity and to assess the potential use of synovial ADC as a diagnostic marker for therapy surveillance.

## Competing interests

The authors declare that they have no competing interests with regard to the presented work.

## Authors’ contributions

HN is the guarantor of the presented work. HN, DH and MB conceived, planned and conducted the study. LE and MP participated in data acquisition and analysis. HM and HG shared in data interpretation and literature review. HK was responsible for developing MRI sequences. All authors participated in drafting and revising the manuscript and approved the submitted version.
